# The effectiveness of non-surgical interventions in athletes with groin pain: a systematic review and meta-analysis

**DOI:** 10.1186/s13102-023-00684-6

**Published:** 2023-07-10

**Authors:** Silvia Lahuerta-Martín, Román Robles-Pérez, Ignacio Hernando-Garijo, Sandra Jiménez-del-Barrio, Héctor Hernández-Lázaro, María Teresa Mingo-Gómez, Luis Ceballos-Laita

**Affiliations:** 1 Department of Surgery Ophthalmology, Otorhinolaryngology and Physiotherapy, University of Valladolid, 42004 Soria, Spain; 2 Clinical Research in Health Sciences Group, University of Valladolid, 42004 Soria, Spain

**Keywords:** Groin pain, Manual therapy, Pain, Range of motion

## Abstract

**Background:**

Groin pain is a common pathology among athletes, presenting pain and a reduced range of motion (ROM) as clinical characteristics. Passive physical therapy (PPT) and exercise therapy (ET) interventions are chosen firstly before surgery. The aim of this systematic review and meta-analysis was: (i) to qualitative review the effects of each non-surgical intervention; (ii) to quantitative compare the effects of PPTs plus ET intervention to ET in isolation in pain intensity, and hip ROM in athletes with groin pain.

**Methods:**

A systematic review and meta-analysis was conducted. Pubmed, PEDro, Web of science, Scopus and Cochrane library were searched. Randomized controlled trials comparing PPT plus ET to ET interventions were included. The methodological quality and risk of bias of the included studies, were assessed with the PEDro scale and the Cochrane risk-of-bias tool. To assess the certainty of evidence the GRADEpro GDT was used. Meta-analyses were conducted using RevMan 5.4 using mean difference analysis to assess the variables pain intensity and hip ROM.

**Results:**

A total of 175 studies was identified from the consulted databases. Five studies were included for systematic- review, from which three studies were meta-analyzed. The methodological quality of the included studies ranged from poor to high. ET compared to PPT plus ET provided statistically significant improvements in pain intensity in the short-term (MD = 2.45; 95% CI 1.11, 3.79; I^2^ :65%). No statistically significant differences between interventions were obtained for hip ROM in the short-term.

**Conclusions:**

The qualitative review showed that PPTs plus ET and ET seem to have positive effects on pain intensity and hip ROM. The quantitative analysis found very low certainty of evidence proposing a positive effect in pain intensity for ET interventions based on hip muscles stretching, compared to PPT combined with ET, in the short-term.

**Supplementary Information:**

The online version contains supplementary material available at 10.1186/s13102-023-00684-6.

## Background

Groin pain is a common problem for recreational and professional athletes characterized by pain or discomfort in the inguinal and/or anterior pelvic region [1]. The complexity of the hip-spine structures contributes to the use of different terms to identify this problem, such as sport hernia, athletic pubalgia, osteitis pubis, biomechanical groin overload, and Gilmore’s groin among others; that are no longer recommended [2]. Therefore, there is an uncertain epidemiological estimation (accounted for 4–19% of all injuries in soccer players), and it is difficult to make an adequate diagnosis and treatment [1, 3].

To clarify this situation, the Doha Agreement Meeting concluded that groin pain could be classified according to the recognizable pattern of symptoms and signs as: (1) defined clinical entities for groin pain such as adductor-related groin pain; (2) hip-related groin pain; or (3) other causes of groin pain [2]. According to the Doha Agreement Meeting, the groin pain symptoms are mainly related to hip joint or the soft tissues surrounding the hip joint. For that reason, the clinical characteristics of athletes with groin pain include pain, lower strength on hip adductor muscles, and reduced hip ROM, especially hip internal rotation (IR) ROM compared to healthy subjects [4–6].

Non-surgical interventions are the first-line recommendation for the management of groin pain [7]. Among non-surgical interventions a wide variety of interventions can be found and divided as: (i) PPT interventions including heat, deep friction massage, electrotherapy, or muscle stretching [8–16]; and (ii) active therapies including different types of ET [11–18]. Despite that, it is not known which type of intervention presents more benefits for athletes with groin pain.

Previous systematic reviews investigated the effects of non-surgical interventions in athletes with groin pain. Nevertheless, these reviews included case reports, retrospective case series, cross-sectional studies, single-cohort studies, and randomized controlled trials (RCTs) involving heterogeneous interventions [19–22]. Based on the scientific literature review, no systematic review and meta-analysis has been carried out showing the effects of PPT and ET and comparing its effectiveness. Thus, the aim of this systematic review and meta-analysis was: (1) to qualitative review the effects of each non-surgical intervention; (2) to quantitative compare the effects of PPTs interventions combined with ET to ET in isolation in pain intensity, and hip ROM in athletes with groin pain.

## Methods

### Study design

The protocol of this systematic review and meta-analysis was registered in the International Prospective Register of Systematic Reviews (PROSPERO) with the registration number CRD42023401039. This systematic review and meta-analysis followed the Preferred Reporting Items for Systematic Reviews and Meta-analysis (PRISMA) statement and Cochrane recommendations [23].

### Search strategy

The bibliographical search was conducted in PubMed (MEDLINE), Physiotherapy Evidence Database (PEDro), Scopus; Cochrane Library, and Web of Science (WOS) from inception to 18 February 2023. The Population, Intervention, Comparison, Outcome and Study design (PICOS) framework was used to define the search strategy. The main keywords used in the search strategy were: groin pain, osteitis pubis, pubalgia, athletic pubalgia, physical therapy modalities, and exercise. The strategies used for each database are shown in Additional file 1. Scopus database was included as a tool for searching grey literature, and a hand search of the reference list of the included studies was performed. Searches were not limited by language.

### Eligibility criteria and study selection

The included studies met the PICOS criteria: (1) Population: athletes diagnosed with any type of groin pain; (2) Intervention: PPT modalities (electrotherapy, thermotherapy, manual therapy techniques, or stretching) combined with ET; (3) Comparison: ET; (4) Outcomes consisted of pain intensity, and hip ROM; (5) Study design: RCTs.

Studies were excluded if they: (1) included participants without groin pain; (2) the intervention was based on surgical approaches or the physical therapy intervention included pharmacological therapies; (3) the outcome variables reported were not the outcomes of interest or were not measured using a valid and reliable instrument.

After searches were retrieved, references were exported to Mendeley desktop, and duplicates were removed. Two assessors independently (LC and SL) assessed the title and abstract of each reference to determine potential eligibility. The same independent assessors assessed potential full texts. A third assessor (SJ) resolved the discrepancies between the two assessors. Two assessors were contacted by e-mail to clarify eligibility criteria. The inter-rater agreement was calculated using the Cohen’s kappa coefficient [24].

### Data extraction

The two assessors (LC and SL) independently extracted the data from the identified studies using the standardized process adapted from the Cochrane Collaboration. Extracted information included: (1) characteristics of the study population; (2) aspects of the intervention performed; (3) outcome measures; (4) results; and (5) follow-up period. The third assessor (SJ) resolved any disagreements. Data were analyzed using a qualitative synthesis and, whenever possible, using a quantitative synthesis (meta-analysis).

### Risk of bias and certainty of the evidence

Two assessors (LC, SL) assessed the quality of the studies using the PEDro scale and the Cochrane Risk-of-Bias tool.

The PEDro scale consists of 11-items following the Delphi List [25]. The first item is related to the external quality of the study but it is not included in the total score [26]. The rest items evaluate credibility, internal consistency and the interpretation of the results [26]. A score below 4 showed “poor” quality, between 5 and 6 showed “fair” quality, and above 7 showed “high” quality [25, 27]. The methodological quality of RCTs can be assessed in a reliable and valid way with the PEDro scale [28, 29].

The Cochrane Risk-of-Bias tool determines the potential bias and the internal validity of the studies and classifies them as “low”, “unclear”, or “high” risk based on 7 criteria [30]. This tool has shown to be reliable for evaluating the quality of the studies and assessing the risk of bias. Funnel plot asymmetry, to assess publication bias in the meta-analyses, was not conducted in this study because the meta-analyses presented did not meet the rule of at least 10 trials.

The GRADEpro GDT was used to assess the certainty of evidence and develop a summary of the findings. This classification categorizes the evidence as “high”, “moderate”, “low”, or “very low” and allows researchers and clinicians to discern the importance of the results. The certainty of evidence for the meta-analysis was downgraded according to the presence of the following: (i) risk of bias (downgraded by one level or two levels if more than 25% or 50% of the participants were from studies with poor or fair methodological quality: lack of allocation concealment, random allocation and/or sample size calculation, participant, and personnel blinding, blinding of outcome assessors), (ii) inconsistency of results (downgraded by one level if there was significant heterogeneity regarding outcome measurement or intervention, or if the I^2^ value was ≥ 50%, and two levels if the I^2^ was ≥ 75%)[31, 32], (iii) indirectness of evidence (downgraded by one level if different populations, interventions, or comparators were included), and (iv) imprecision (downgraded by one level if fewer than 100 participants were included in the comparison, and two levels when the sample sizes were ≤ 30 individuals) [32–34]. Single randomized trials were considered inconsistent and imprecise and provided “low certainty” evidence. This could be further downgraded to “very low” certainty evidence if there was also a high risk of bias [30, 35].

### Data synthesis and analysis

The quantitative synthesis of the results was carried out according to the considered outcomes: pain intensity and hip ROM. Separate analyses were performed for each outcome variable. Mean, standard deviation (SD) and sample size at each time point were extracted for each group. Outcomes were analyzed based on the post-intervention means and SDs by calculating the mean difference (MD) when studies used the same scale or the standardized mean difference (SMD) with 95% confidence interval (CI), when different tools were used across studies. Significance was set at a *P* value < 0.05. The minimum clinically important change on pain intensity, and hip IR ROM have not been described for patients with groin pain. Data were combined for meta-analysis using a minimum of two trials assessed as clinically homogeneous. Trials were considered clinically homogeneous if there was a common intervention and outcome. Random-effect meta-analysis was performed when the combination of intervention effects could incorporate an assumption that the studies are not all estimating the same intervention effect [36]. A researcher analyzed data of interest outcomes using RevMan 5.4 software.

## Results

### Literature search and screening

**Fig. 1:**
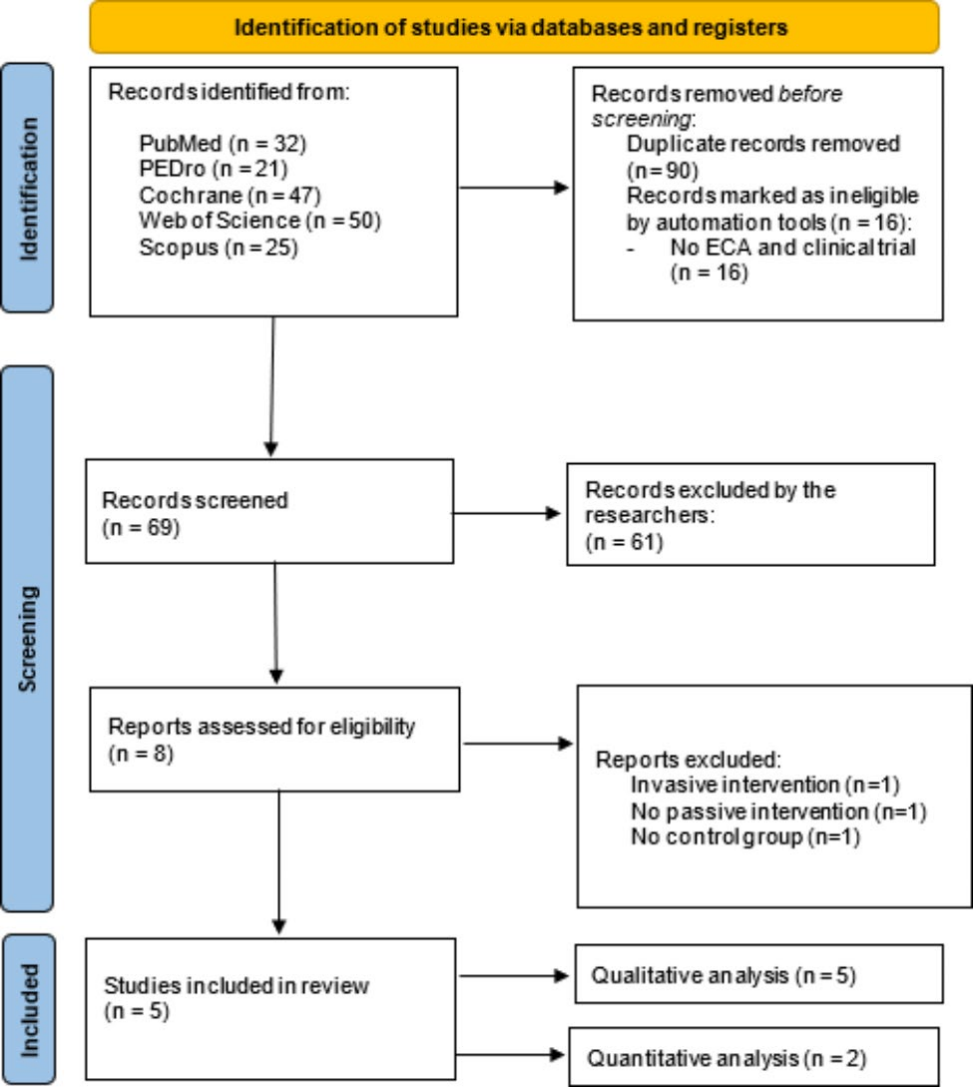
PRISMA flow chart diagram

A total of 175 studies was identified from the consulted databases. After removing duplicates and screening by title and abstract, 8 studies were full-text reviewed. Three studies were excluded after the full-text review. One study applied an invasive technique [37], other did not include a PPT intervention [18], and the other did not include a comparison group [38]. Finally, 5 studies met the eligibility criteria and were included in the qualitative synthesis. In the quantitative synthesis 3 studies were included, and 2 were excluded for presenting non-comparable data in the results Sects. [12, 16]. Four of the included studies were primary studies [13–16] and one was a long-term analysis of the primary RCT [12]. The description of the selection process is shown in the PRISMA flowchart diagram (Fig. 1). The agreement between reviewers was calculated by kappa with a value of 0.99.

### Characteristics of included studies

A total of 5 RCTs was included comprising 196 athletes with groin pain (the population from the secondary analysis of the RCT was not added to the total sample). The sample size ranged from 40 to 68 patients across the studies.

All the studies included athletes with groin pain. Soccer athletes were present in all the studies [13–16]. Holmich et al. [16] and Weir et al. [13] also included athletes from other sports. The athletes included were diagnosed as long-standing adductor related groin pain in 2 studies [13, 16], adductor-related groin pain in one study [15], and sports hernia in other study [14]. The sociodemographic and clinical characteristics of the participants in each study are shown in Table 1.

**Table 1:** Sociodemographic and clinical characteristics of the included studies

Autor (year)	N (sex ratio)	Mean age (SD)	Sports	PPT group	ET group	Outcomes	Main Results	Follow-up
Holmich et al. (1999)	68 PPT: 34 ET: 34	PPT: 30 (21–50) ET: 30 (20–50)	Soccer Running Rugby Tennis Handball Badminton Basketball Ice hockey Horse riding	Electrotherapy + manual therapy + stretching + return to running program	First 2 weeks: static adduction, abdominal sit-ups, abdominal sit-up and hip flexion, balance training, one-foot exercises. From 3er week: leg adduction, low-back extension, one-leg weight-pulling abduction, abdominal sit-ups, balance training, skating movements + return to running program	Pain Hip ABD ROM	ET: 79% returned to sports activity without pain. PT: 14% returned to sports activity without pain. ↑ hip ABD ROM in both groups. No between-groups differences in hip ABD ROM.	Holmich et al. (2011). 8-to 12-year follow-up. The beneficial effects of ET in pain were lasting.
Weir et al. (2011)	48 PPT: 26 ET: 22	PPT: 28.7 (8.2) ET: 27.4 (7.3)	Soccer Rugby Squash Running Hockey Skating Other sports	Muscle heat + manual therapy + stretching + return to running program	Holmich et al. protocol + “return to running” program	Pain (VAS) hip ROM	↑ pain in both groups. No between-groups differences in any variable.	No data
Abouelnaga & Aboelnour (2019)	40 PPT: 20 ET: 20	PPT: 26.75 (3.02) ET: 26.2 (2.94)	Soccer	Muscle heat + electrotherapy + manual therapy + stretching + return to running program	Strengthening of the abdominal and hip muscles + core stabilization + balancing exercises + return to running program	Pain (VAS) Hip IR ROM Hip ER ROM	↑pain, IR and ER ROM in both groups. ↑ pain in ET vs. PPT.	No data
Qashees et al. (2021)	40 PPT: 20 ET: 20	PPT: 21.5 (3.4) ET 21.9 (4.2)	Soccer	Manual therapy + passive stretching + hip abductor and adductor muscles strengthening	Hip abductor and adductor muscles strengthening	Hip IR ROM	↑IR ROM in both groups. ↑ IR ROM in PPT vs. ET.	No data

Abbreviation: N: Sample size; SD: Standard deviation; PPT: passive physical therapy; ET: exercise therapy; VAS: visual analogue scale; IR: internal rotation; ER: external rotation; ROM: range of motion; ABD: abduction

↑ Statistically significant improvement

The PPT groups included muscle heat, electrotherapy, manual therapy, transverse friction massage, and stretching techniques. Three of them were combined with a return to running program [13, 14, 16], and one with hip muscle strengthening [15]. The ET groups included hip strengthening programs. Two of them followed the Holmich et al. protocol [13, 16].

The most used frequency was 3 sessions a week and the total number of weeks varied across the studies from 6 to 24 weeks. The description of the interventions, the duration of the sessions, the number of sessions per week, and the total number of sessions are presented in Table 2.

**Table 2:** Characteristics of the interventions

Author	PPT group	ET group	Frequency (sessions a week)	Length (weeks)	Total sessions
Holmich et al. (1999) (2011)	Electrotherapy (laser and transcutaneous electrical nerve stimulation) + transverse friction massage + adductor, hamstrings and hip flexor muscles stretching + return to running program	First 2 weeks: static adduction, abdominal sit-ups, abdominal sit-up and hip flexion, balance training, one foot-exercises. From 3er week: leg adduction, low back extension, one leg weight-pulling abduction, abdominal sit-ups, balance training, skating movements. + return to running program	PPT: 3 ET: 2	8–12	NR
Weir et al. (2011)	Muscle heat + manual therapy + adductor stretching + return running program after 14 days of stretching	Holmich et al. protocol + return to running program	3	16–24	NR
Ahmed Abouelnaga & Hassan Aboelnour (2019)	Muscle heat + electrotherapy (transcutaneous electrical nerve stimulation) + transverse friction massage + pelvic and hip mobilizations + adductor, hamstrings and hip flexor muscles stretching + return to running program.	Core stabilization + hip and abdominal strengthening exercises + balancing exercises + return to running program	3	8	24
Qashees et al. (2021)	Manual therapy + Adductor muscles stretching + adductor muscles strengthening	Adductor muscles stretching + adductor muscles strengthening	3	6	18

Abbreviation: PPT: passive physical therapy; ET: exercise therapy; NR: Not reported

### Outcome measures

The outcomes considered in this systematic review and meta-analysis were pain intensity and hip ROM. Three studies assessed pain intensity using the visual analogue scale (VAS) [13–15] and all measured hip ROM. Three measured hip IR ROM [13–15], one hip external rotation (ER) ROM [14], and one hip abduction ROM [16].

All the studies assessed the outcome variables at baseline and after the intervention (short-term) [13–16]. Concerning the follow-up periods, the secondary analysis of Holmich et al.[16] assessed the long-term at 8-to 12-year follow-up [12].

### Study quality and risk of bias

The methodological quality and risk of bias of the included studies were assessed using the PEDro scale and the Cochrane risk-of-bias tool. According to the PEDro scale, there were no studies with participant and therapist blinding. All studies presented baseline comparability and between-group statistical comparisons. Two studies presented high methodological quality [13, 16], two studies presented fair quality [12, 14], and one study presented poor quality [15]. The results for the PEDro scale are shown in Table 3.

**Table 3:** PEDro scale score

Author	Items											
	1	2	3	4	5	6	7	8	9	10	11	Total
Holmich et al. (1999)	Y	Y	Y	Y	N	N	Y	Y	Y	Y	Y	8/10
Holmich et al. (2011)	N	Y	N	Y	N	N	Y	N	N	Y	Y	5/10
Weir et al. (2011)	Y	Y	Y	Y	N	N	Y	Y	N	Y	Y	7/10
Abouelnaga & Aboelnour (2019)	N	Y	Y	Y	N	N	N	Y	N	Y	Y	6/10
Qashees et al. (2021)	Y	N	N	Y	N	N	N	Y	Y	Y	N	4/10

Abbreviations: N: no; Y: yes

**Fig. 2:**
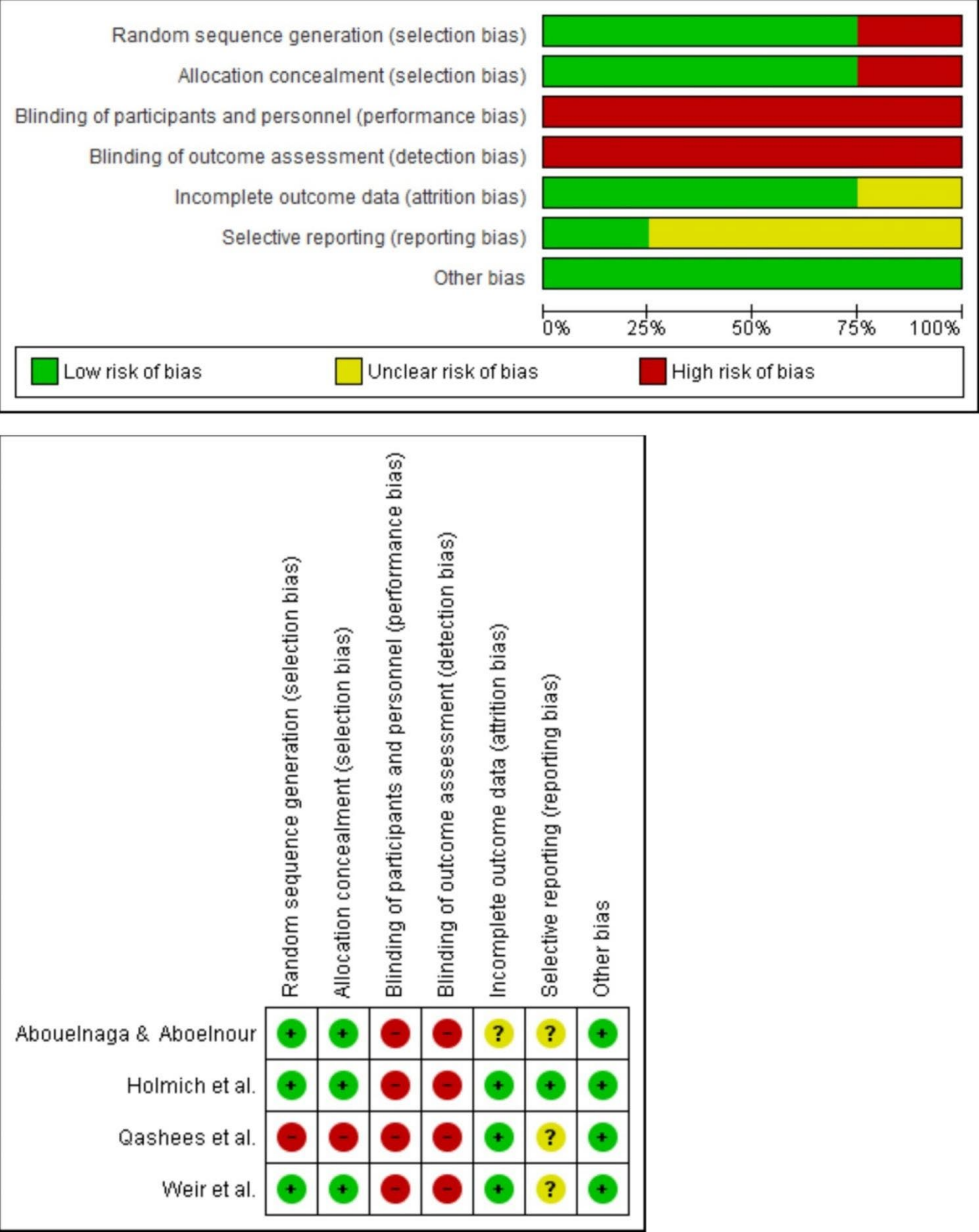
Cochrane risk-of-bias tool

According to the Cochrane risk-of-bias tool all the RCTs included in this review showed a high risk of performance and detection bias. Most of the studies performed correctly the random sequence generation, and the concealment allocation. No study blinded the participants or therapist (this item is expected in conservative non-pharmacological interventions). The Cochrane risk-of-bias tool results are shown in Fig. 2.

### Certainty of evidence

The certainty of evidence was assessed with GRADEpro GDT. The overall certainty of evidence was rated as very low for pain intensity and hip ROM in all studies in the short-term. The GRADE analyses are shown in Additional file 2.

### Synthesis of results

#### Pain intensity

Three studies were included in the qualitative synthesis. Two studies found that both interventions were effective for improving pain intensity in patients with groin pain [13, 14]. In the between-groups analysis, two studies showed that ET based on hip muscles strengthening was more effective for decreasing pain intensity compared to PPT plus return to running program [14, 16] in the short-term. The secondary analysis of Holmich et al. [12] concluded that the improvement in pain intensity was maintained at 8- to 12- year of follow-up.

**Fig. 3:**
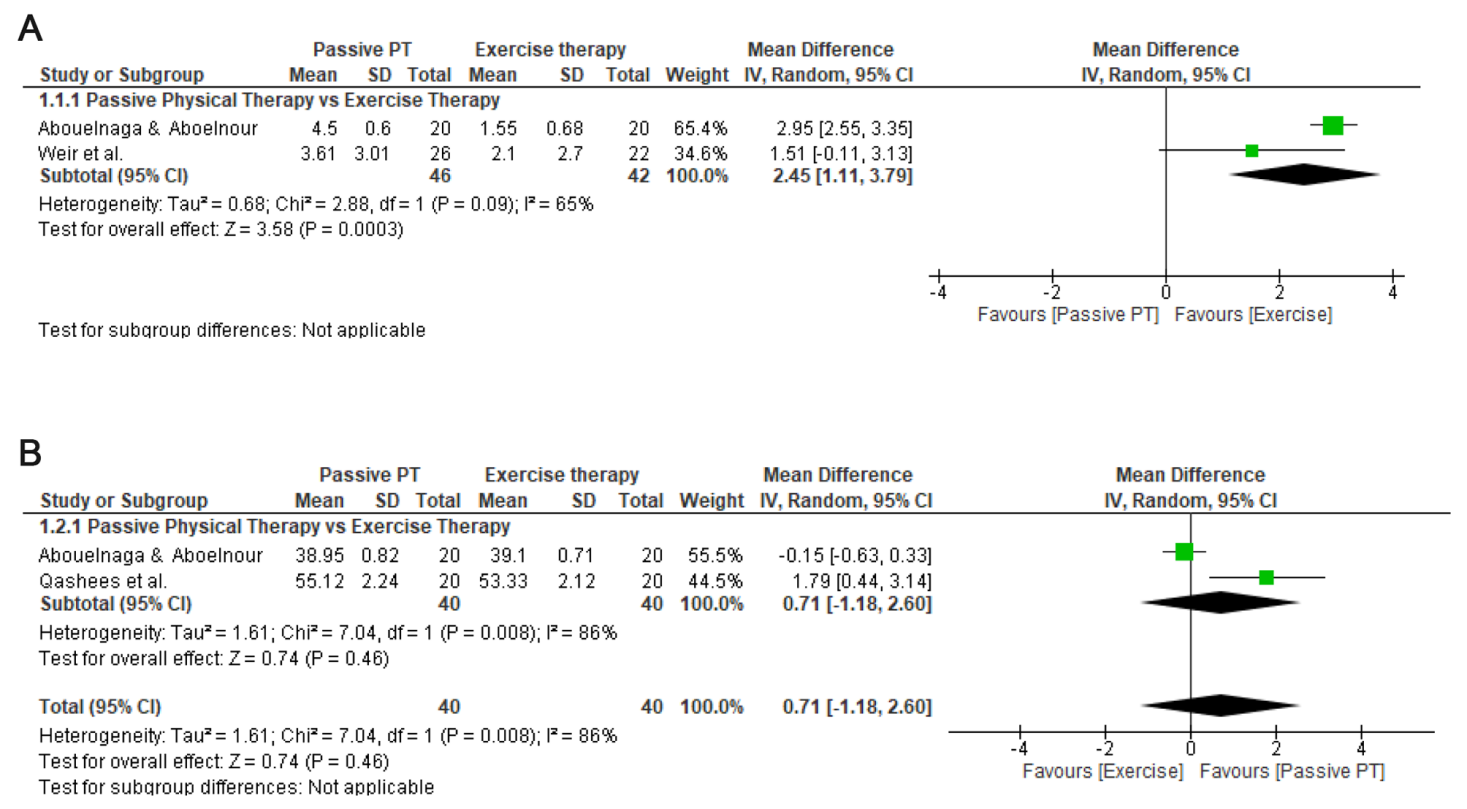
**A** Forest plot of pain intensity in the short-term. **B**. Forest plot of hip IR ROM in the short-term

Two studies were included in the quantitative synthesis [13, 14]. Very low certainty evidence showed that ET based on hip muscles strengthening provided statistically significant improvements in pain intensity in the short-term compared to PPT plus a return to running program (MD = 2.45; 95% CI 1.11, 3.79; I^2^ :65%; 2 studies, 88 participants) (Fig. 3A).

### Hip ROM

Four studies were included in the qualitative synthesis. Three studies assessed hip IR ROM [13–15], one assessed hip ER ROM [14], and one assessed hip abduction ROM [12, 16]. Three studies found that both interventions were effective for improving hip ROM in athletes with groin pain [14–16]. In the between-groups analysis, no between-groups differences were found in any study in the short-term. Only Qashees et al. [15] found statistically significant improvements in hip IR ROM in favor of the PPT group.

Two studies were included in the quantitative synthesis [14, 15]. Very low certainty evidence showed no statistically significant differences between both interventions (MD = 0.71; 95% CI -1.18, 2.60; I^2^ :86%; 2 studies, 80 participants) (Fig. 3B).

## Discussion

The results of this systematic review and meta-analysis found that most of the studies concluded that PPTs plus ET or ET seem to have positive effects on pain intensity and hip ROM. After the quantitative synthesis, very low certainty of evidence suggest that ET is more beneficial than PPTs for improving pain intensity, but not statistically significant differences between groups for hip ROM. According to the certainty of evidence summary, the level of evidence was downgraded to very low due to serious and very serious records in risk of bias, inconsistency, and imprecision. For risk of bias the certainty of evidence was downgraded because of no study had blinded participants, therapists and/or outcome assessment; and the allocation concealment was not fulfilled for all included studies. Related to inconsistency, reasons for downgraded were the lack of homogeneity of interventions and I^2^ values higher than 50% or 75% for both studied variables. Imprecision assessment was downgraded to serious for both pain intensity and hip ROM due to sample sizes of the studies had less than 100 participants to compare.

The methodological quality of the included RCTs in this systematic review and meta-analysis ranged from poor to high according to the PEDro scale. The most common flaws were the absence of allocation concealment, blinding of participants, blinding of therapists and intention-to -treat analysis. It is important to take into account that blinding of therapists is not possible in physical therapy interventions [39]. The PEDro scale results were corroborated with those produced by Cochrane risk of bias tool in which there was highlighted high risk of performance and detection bias and 25% selection bias.

PPT and ET interventions had been proposed for the treatment of different types of groin pain obtaining positive results [20, 21]. However, these systematic reviews did not include RTCs and the studies analyzed were level 4 evidence [20, 21] or included a mixture RCTs and level 4 evidence studies [19, 22]. To the best of our knowledge this is the first systematic review and meta-analysis that includes only level 1 evidence studies investigating the effects of PPTs combined with ET and compared to ET in isolation. Moreover, this is the first meta-analysis carried out about this topic, attaching analyses of methodological quality and certainty of evidence.

The systematic review and meta-analysis included 5 studies for qualitative analysis [12–16], and 3 of them could be included for quantitative analysis [13–15]. The results achieved showed that ET produced a significant decrease in pain intensity in the short term (MD = 2.45) compared to PPT plus ET. For hip ROM, the results obtained were not significant between interventions in the short-term (MD = 0.71). These results could be explained for different reasons. First of all, there was a little number of studies included for meta-analysis and the interventions were heterogeneous between studies. For pain intensity, the PPT intervention of Abouelnaga & Aboelnour [14] included 5 different techniques (heat, transverse friction massage, TENS, mobilization techniques and stretching exercises), whereas Weir et al. [13] included muscle heat, manual therapy and adductor stretching. Moreover, ET differed between the two studies. The same occurred for hip ROM, in which the compared studies Abouelnaga & Aboelnour [14] and Qashees et al. [15] applied different interventions both in PPT and ET. Another reason to take the results carefully is that PPT interventions were generally composed by various techniques. Thus, it might be not possible to stablish which of them had a better response in the patients. Additionally, there was only one study that assessed the long-term effects of one intervention, but it was not possible to include it for meta-analysis [12]. Therefore, it was difficult to stablish the effectiveness of both PPT or ET interventions.

It is important to point out that the population included in the studies had different diagnostics like groin hernia [14], long-standing adductor-related groin pain [13] or adductor-related groin pain [15]. It will be important that future research stablish correctly the groin pain entity to contribute to different specific interventions and its generalization to an specific population. For these reasons, there was no posible comparison with previous meta- analyses.

From a clinical perspective, groin pain is a complex pathology which is difficult to diagnose and to manage. The current evidence suggests that multimodal interventions including passive and/or active techniques produce improvements in pain intensity and hip ROM in the short-term. However, the lack of unification of the diagnostic terms does not allow us to know which intervention is most beneficial for each type of patient with groin pain. In addition, the application of various techniques in the intervention groups does not allow us to know which techniques produce the best effects in these patients.

This study has some limitations. The search strategy was limited to specific databases, so other potential databases were omitted. The heterogeneity of the included studies as well as the small samples sizes may complicate the interpretation of the results. With relation to the methodological quality, it could be of great interest the correct blinding of participants and therapists; and the management of the results. Further investigation must be carried out to clarify short- and long-term effects and to specify the application parameters and the role of PPT interventions as isolate specific techniques.

The strengths of our systematic review and meta-analysis include a comprehensive literature search, methodological rigor, data extraction, rigorous statistical analysis, and the inclusion of only RCTs. This study may be integrated into the evidence-based medicine framework because it includes data from RCTs (level 1b evidence) and uses GRADE recommendations for its conclusions.

## Conclusion

The qualitative synthesis of the included studies showed that both PPTs plus ET or ET seem to have positive effects on pain intensity and hip ROM in patients with groin pain. The quantitative analysis found very low certainty of evidence proposing a positive effect in pain intensity for ET interventions based on hip muscles stretching compared to PPT combined with ET, in the short-term. The results should be interpreted with caution due to the small number of included studies.

## Electronic supplementary material

Below is the link to the electronic supplementary material.

Supplementary Material 1

Supplementary Material 2

## Data Availability

All data generated or analysed during this study are included in this published article and additional files.

## Abbreviations

ROM: Range of Motion
PPT: Passive Physical Therapy
ET: Exercise Therapy
RCT: Randomized Controlled Trial
IR: Internal Rotation
SD: Standard Deviation
SMD: Standardized Main Difference
MD: Mean Difference
CI: Confidence Interval
VAS: Visual Analogue Scale
ER: External Rotation
N: Sample size
NR: Not reported
