# Supplementary material for: The effectiveness of non-surgical interventions in athletes with groin pain: a systematic review and meta-analysis
Source: BMC Sports Sci Med Rehabil. 2023 Jul 10;15:81. doi: 10.1186/s13102-023-00684-6 (PMC10332077; doi:10.1186/s13102-023-00684-6)
Supplement: Supplementary file 1 — Supplementary Material 1 [file 13102_2023_684_MOESM1_ESM.pdf]

Additional file 1. Search strategy

PubMed

("groin pain" OR "osteitis pubis" OR pubalgia OR "athletic pubalgia") AND (physical therapy modalities [MeSH] OR exercise [MeSH] OR "manual therapy" OR "physical therapy" OR physiotherapy) AND (pain [MeSH] OR "range of motion" OR "range of movement" OR "physical function" OR "functional capacity" OR "physical performance") AND ("trial" OR "clinical trial" OR "randomized clinical trial" OR "randomised clinical trial")

Date: 18/02/2023

Studies retrieved: 32

PEDro

groin pain

Studies retrieved: 19

Osteitis pubis

Studies retrieved: 1

Pubalgia

Studies retrieved: 1

Date: 18/02/2023

COCHRANE Library

("groin pain" OR "osteitis pubis" OR pubalgia OR "athletic pubalgia") AND (physical therapy modalities OR exercise OR "manual therapy" OR "physical therapy" OR physiotherapy) AND (pain OR "range of motion" OR "range of movement" OR "physical

function" OR "functional capacity" OR "physical performance") AND ("trial" OR "clinical trial" OR "randomized clinical trial" OR "randomised clinical trial")

Date: 18/02/2023

Studies retrieved: 46

WEB OF SCIENCES

("groin pain" OR "osteitis pubis" OR pubalgia OR "athletic pubalgia") AND (physical therapy modalities OR exercise OR "manual therapy" OR "physical therapy" OR physiotherapy) AND (pain OR "range of motion" OR "range of movement" OR "physical function" OR "functional capacity" OR "physical performance") AND ("trial" OR "clinical trial" OR "randomized clinical trial" OR "randomised clinical trial")

Date: 18/02/2023

Studies retrieved: 50

SCOPUS

TITLE-ABS-KEY (("groin pain" OR "osteitis pubis" OR pubalgia OR "athletic pubalgia") AND (physical AND therapy AND modalities OR exercise OR "manual therapy" OR "physical therapy" OR physiotherapy) AND (pain OR "range of motion" OR "range of movement" OR "physical function" OR "functional capacity" OR "physical performance") AND ("trial" OR "clinical trial" OR "randomized clinical trial" OR "randomised clinical trial"))

Date: 18/02/2023

Studies retrieved: 25
