# Supplementary material for: The effectiveness of non-surgical interventions in athletes with groin pain: a systematic review and meta-analysis
Source: BMC Sports Sci Med Rehabil. 2023 Jul 10;15:81. doi: 10.1186/s13102-023-00684-6 (PMC10332077; doi:10.1186/s13102-023-00684-6)
Supplement: Supplementary file 2 — Supplementary Material 2 [file 13102_2023_684_MOESM2_ESM.pdf]

Additional file 2. GRADE. Summary of evidence of the results according to their certainty.

Certainty assessment							Number of patients		Effect		Certainty
Number of studies	Study Design	Risk of bias	Inconsistency	Indirect evidence	Imprecision	Other considerations	[Pasive PT]	[Exercise therapy]	Relative (95% CI)	Absolute (95% CI)	
**Pain intensity (evaluated with: VAS; Scale from: 0 to 10)**											
2	RCT	Serious[a]	Serious[b]	Not serious	Serious[c]	None	46	42	-	MD 2.45 **higher.** (1.11 higher. to 3.79 higher.)	⊕◯◯◯ Very low
**Hip IR ROM**											
2	RCT	Very serious	Very serious	Not serious	Serious	None	40	40	-	MD 0.71 **higher.** (1.18 higher. to 2.60 higher.)	⊕◯◯◯ Very low

RCT: randomized clinical trial; **CI:** confidence interval; **MD:** mean difference.

EXPLANATIONS

a. No blinding participants, therapists and/or assessors.
b. No homogeneity of interventions.
c. Less than 100 participants.
